# Reduced complement of dopaminergic neurons in the substantia nigra pars compacta of mice with a constitutive “low footprint” genetic knockout of alpha-synuclein

**DOI:** 10.1186/s13041-020-00613-5

**Published:** 2020-05-11

**Authors:** Valeria V. Goloborshcheva, Kirill D. Chaprov, Ekaterina V. Teterina, Ruslan Ovchinnikov, Vladimir L. Buchman

**Affiliations:** 1 School of Biosciences, Cardiff University, Museum Avenue, Cardiff, CF10 3AX UK; 2 Institute of Physiologically Active Compounds Russian Academy of Sciences (IPAC RAS), 1 Severniy proezd, Chernogolovka, Moscow Region, Russian Federation 142432; 3 Pirogov Russian National Research Medical University, Ostrovitianov Str., 1, Moscow, Russian Federation 117997

**Keywords:** Dopaminergic neurons, Substantia nigra, Striatum, Alpha-synuclein, Null mutant mice

## Abstract

Previous studies of the alpha-synuclein null mutant mice on the C57Bl6 genetic background have revealed reduced number of dopaminergic neurons in their substantia nigra pars compacta (SNpc). However, the presence in genomes of the studied mouse lines of additional genetic modifications that affect expression of genes located in a close proximity to the alpha-synuclein-encoding *Snca* gene makes these data open to various interpretations. To unambiguously demonstrate that the absence of alpha-synuclein is the primary cause of the observed deficit of dopaminergic neurons, we employed a recently produced constituent alpha-synuclein knockout mouse line B6(Cg)-*Snca*^*tm1.2Vlb*^/J. The only modification introduced to the genome of these mice is a substitution of the first coding exon and adjusted short intronic fragments of the *Snca* gene by a single loxP site. We compared the number of dopaminergic neurons in the SNpc of this line, previously studied B6(Cg)-*Snca*^*tm1Rosl*^/J line and wild type littermate mice. A similar decrease was observed in both knockout lines when compared with wild type mice. In a recently published study we revealed no loss of dopaminergic neurons following conditional inactivation of the *Snca* gene in neurons of adult mice. Taken together, these results strongly suggest that alpha-synuclein is required for efficient survival or maturation of dopaminergic neurons in the developing SNpc but is dispensable for survival of mature SNpc dopaminergic neurons.

## Background

Gain-of-function of alpha-synuclein has been strongly linked to aetiology and pathogenesis of Parkinson’s and several other neurodegenerative diseases (recent advances and perspectives are summarised in Ref [1]). However, despite a growing body of evidence that suggests its involvement in many important molecular processes in healthy neurons, predominantly in presynaptic terminals (for recent review see Ref [2]), it is not clear if alpha-synuclein was required for survival and/or maturation of neurons. Previous studies have demonstrated that at least on the C57Bl6 genetic background, adult mice lacking alpha-synuclein as the result of a naturally occurred mutation or targeted inactivation of the encoded gene have reduced complement of dopaminergic neurons in their SNpc [3–7]. This deficiency becomes already evident in E12.5 embryonic brains and is not progressive [3, 4, 7]. An uncertainty in interpretation of these results is due to a possibility that some additional genetic modifications present in both mouse lines used in these studies, rather than the absent of alpha-synuclein per se, could potentially cause partial loss of dopaminergic neurons. A naturally occurred deletion in the genome of Harlan UK C57Bl6 mice spans over approximately 350 kilobases, which causes the loss of function not only of alpha-synuclein encoded *Snca*, but also of several other genes [8]. Mice of B6(Cg)-*Snca*^*tm1Rosl*^/J line [9, 10] were produced by homologous recombination that resulted in a substitution of *Snca* exons by a *neo* expression cassette, which presence dramatically activate neuronal expression of a neighbouring *Mmrn1* gene encoding multimerin 1, a protein whose function in the nervous system is enigmatic (Additional file 1). To avoid any disparity in the interpretation of data obtained in different mouse models and reaffirm that the loss of alpha-synuclein is the primary cause of the deficit of dopaminergic neurons in the substantia nigra of null mutant mice, we employed a line of mice with a “clean knockout” of alpha-synuclein. This B6(Cg)-*Snca*^*tm1.2Vlb*^/J line has recently been produced in our laboratory by Cre-driven recombination in the genome of “floxed” and *neo*-free mice of B6(Cg)-*Snca*^*tm1.1Vlb*^/J line followed by crosses with C57Bl6J mice to eliminate the CMV-Cre transgenic cassette. Therefore, the only foreign DNA sequence remaining in the genome of B6(Cg)-*Snca*^*tm1.2Vlb*^/J mice is a single loxP site substituting the first coding exon of *Snca* gene and some adjusted short intronic sequences. This genetic modification completely abolishes production of alpha-synuclein in homozygous animals [11].

## Methods

Null mutant and wild type littermates were produced by intercrossing of heterozygous animals and genotyped as described elsewhere [9, 10]. Brains of 4-month old male mice we fixed, histological sections prepared and immunostained with antibody against tyrosine hydroxylase (TH, mouse monoclonal antibody, clone TH-2, Sigma diluted 1:1000); TH-positive neurons in the SNpc were stereologically counted as described previously [3, 4, 6].

## Results and discussion

**Fig. 1:**
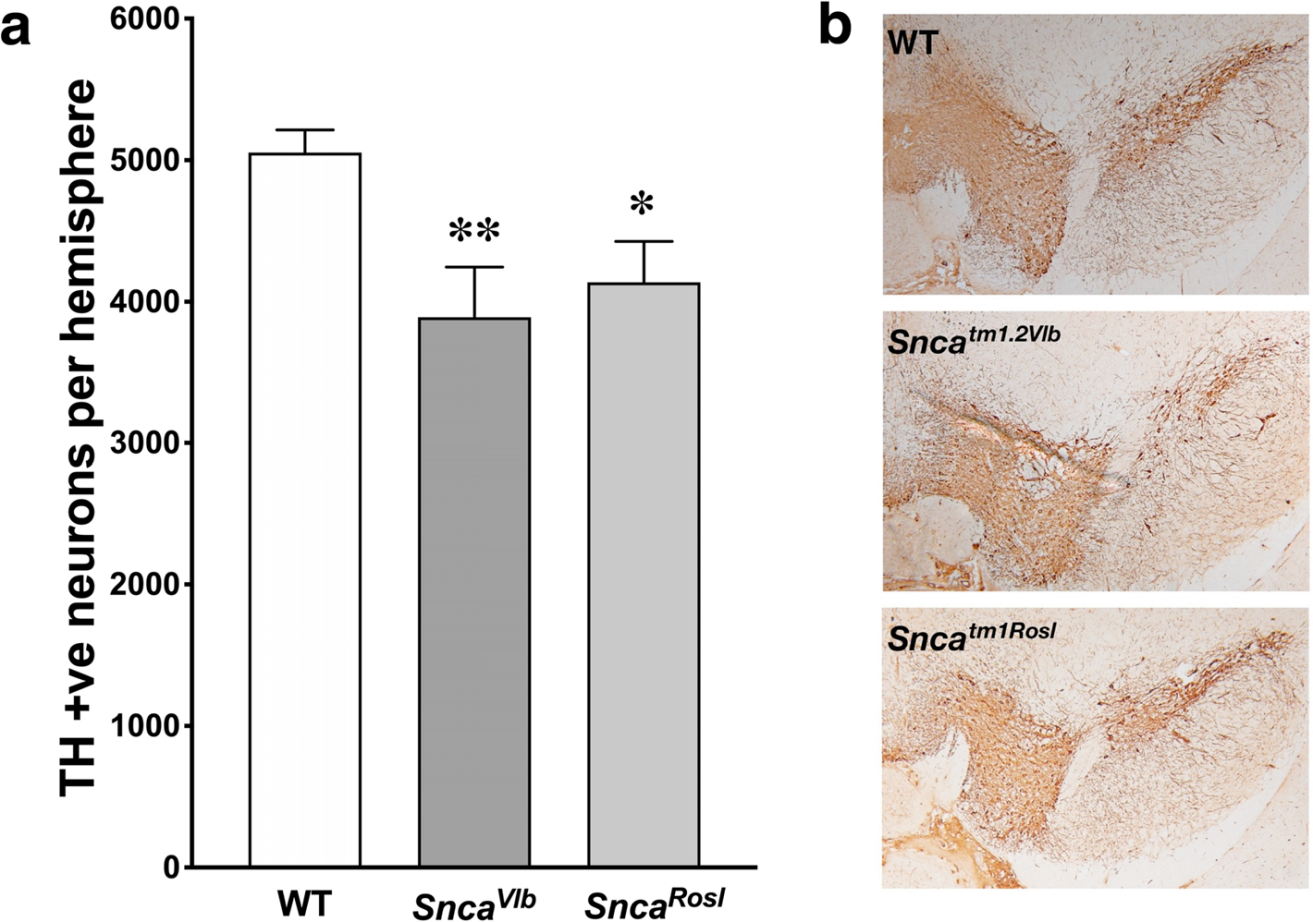
Similar decrease in the number of dopaminergic neurons in SNpc of two alpha-synuclein knockout lines. a) The number of TH-positive neurons in the SNpc of 4-month old wild type (WT), B6(Cg)- *Snca*^*tm1.2Vlb*^/J alpha-synuclein null (*Snca*^*Vlb*^) and B6(Cg)-*Snca*^*tm1Rosl*^/J alpha-synuclein null (*Snca*^*Rosl*^) mice. Bar charts show mean ± SEM of numbers of stereologically counted neurons in SNpc of 8 animals for each genotype. Statistical analysis using one-way ANOVA with Dunnett’s multiple comparisons test revealed significant difference between WT and each of null mutant mouse groups (F (2, 45) = 4.854; *p* = 0.0123; **p = 0.0094; * *p* = 0.0451), whereas the difference between two null mutant mouse groups was not significant (*p* = 0.5329). b) Representative images of brain sections through SNpc region immunostained with antibody against TH

Our morphometric analysis revealed that the total number of dopaminergic neurons in the SNpc of adult B6(Cg)-*Snca*^*tm1Rosl*^/J mice was 18 ± 5.7% lower than in the SNpc of wild type mice (Fig. 1, an additional Excel file, Additional file 2, shows raw count data). This is consistent with the previously reported data for this line as well as for Harlan UK C57Bl6 alpha-synuclein null mutant line [3–6]. Importantly, a similar reduction (23 ± 7.0%) was found in the SNpc of a new “clean knockout” B6(Cg)-*Snca*^*tm1.2Vlb*^/J line (Fig. 1). The latter result clearly implies that dopaminergic neuron deficit observed in studied alpha-synuclein null mutant mouse lines is indeed caused by the lack of alpha-synuclein.

In another recent study we have demonstrated that inactivation of *Snca* gene by the same genetic modification as in B6(Cg)-*Snca*^*tm1.2Vlb*^/J line but induced by Cre recombination in neurons of adult or ageing B6(Cg)-*Snca*^*tm1.1Vlb*^/J animals, caused complete depletion of alpha-synuclein from the dorsal striatum, a brain region where presynaptic terminals (i.e. sites of the predominant localisation of this protein) of the SNpc dopaminergic neurons are located. However, such a late-onset depletion did not lead to any loss of SNpc dopaminergic neurons [12]. Taken together, results of our two studies strongly suggest that alpha-synuclein is required for efficient survival or maturation of dopaminergic neurons in the developing SNpc but is dispensable for survival of mature SNpc dopaminergic neurons. It is also cannot be currently excluded that a certain population of developing dopaminergic neurons is particularly sensitive to the absence of alpha-synuclein.

## Supplementary information

**Additional file 1 **Expression levels of *Mmrn1* mRNA in the cerebral cortex of wild type and synuclein null mutant mice. Bar chart shows relatives level of *Mmrn1* mRNA in the cerebral cortex of the wild type (WT), B6(Cg)- *Snca*^*tm1.2Vlb*^/J alpha-synuclein null (*Snca*^*Vlb*^) and B6(Cg)-*Snca*^*tm1Rosl*^/J alpha-synuclein null (*Snca*^*Rosl*^) mice estimated by real-time quantitative RT-PCR.

**Additional file 2.** Number of TH-positive neurons in SNpc of wild type and synuclein null mutant mice. Raw data for neurons number counts in the left and right SNpc of individual animals used in the study.

## Data Availability

All data generated or analysed during this study are included in this published article [in its supplementary information file].

## Abbreviations

SNpc: Substantia nigra pars compacta
TH: Tyrosine hydroxylase
