# Supplementary figures and images for: Reduced complement of dopaminergic neurons in the substantia nigra pars compacta of mice with a constitutive “low footprint” genetic knockout of alpha-synuclein
Source: Mol Brain. 2020 May 11;13:75. doi: 10.1186/s13041-020-00613-5 (PMC7216632; doi:10.1186/s13041-020-00613-5)

# Mmrn1

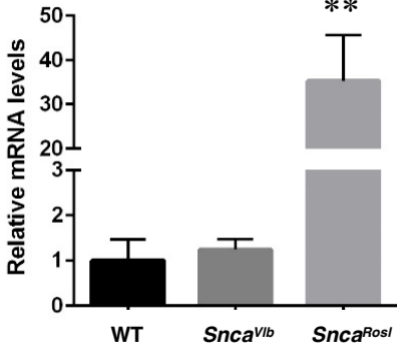

Supplement: Supplementary file 1 — Additional file 1 Expression levels of Mmrn1 mRNA in the cerebral cortex of wild type and synuclein null mutant mice. Bar chart shows relatives level of Mmrn1 mRNA in the cerebral cortex of the wild type (WT), B6(Cg)- Sncatm1.2Vlb/J alpha-synuclein null (SncaVlb) and B6(Cg)-Sncatm1Rosl/J alpha-synuclein null (SncaRosl) mice estimated by real-time quantitative RT-PCR. [file 13041_2020_613_MOESM1_ESM.pdf]
